# A challenging differential diagnosis - Leber’s Hereditary Optic Neuropathy

**DOI:** 10.22336/rjo.2024.13

**Published:** 2024

**Authors:** Raluca Eugenia Iorga, Răzvana Sorina Munteanu-Dănulescu, Ciprian Danielescu

**Affiliations:** * “Grigore T. Popa” University of Medicine and Pharmacy, Faculty of Medicine, Iaşi, Romania; ** Department of Ophthalmology, “N. Oblu” Clinical Emergency Hospital, Iași, Romania; *** Department of Vitreoretina services, MM Joshi Eye Institute, Karnataka, India

**Keywords:** Leber’s hereditary optic neuropathy, optic coherence tomography, retinal ganglion cell layer, optic atrophy, idebenone

## Abstract

Leber’s hereditary optic neuropathy (LHON) is the most common maternally inherited disease linked to mitochondrial DNA (mtDNA). The patients present with subacute asymmetric bilateral vision loss. Approximately 95% of the LHON cases are caused by m.3460G>A (*MTND1*), m.11778G>A (*MTND4*), and m.14484T>C (*MTND6*) mutations. The hallmark of hereditary optic neuropathies determined by mitochondrial dysfunction is the vulnerability and degeneration of retinal ganglion cells (RGC). We present the case of a 28-year-old man who came to our clinic complaining of a subacute decrease in visual acuity of his left eye. From his medical history, we found out that one month before he had the same symptoms in the right eye. From the family history, we noted that an uncle has had vision problems since childhood. We carried out complete blood tests, including specific antibodies for autoimmune and infectious diseases. Laboratory tests and MRI were within normal limits. A blood test of the mtDNA showed the presence of 11778 G>A mutation on the mtND6 gene. The medical history, the fundus appearance, the OCT, and the paraclinical investigations, made us diagnose our patient with Leber’s hereditary optic neuropathy. As soon as possible, we began the treatment with systemic idebenone, 900 mg/day. We examined the patient 2, 6, and 10 weeks after initiating the treatment.

**Abbreviations:** LHON = Leber’s Hereditary Optic Neuropathy, mtDNA = mitochondrial DNA, VA = visual acuity, RE = right eye, LE = left eye, OCT = Optical coherence tomography, pRNFL = peripapillary retinal nerve fiber layer, GCL = retinal ganglion cells layer, MRI = magnetic resonance imaging, VEP = visual evoked potentials, VEP IT = VEP implicit time, VEP A = VEP amplitude

## Introduction

Hereditary optic neuropathies are a group of genetically determined diseases that affect the ganglion cells and the optic nerve [**1**]. Leber’s Hereditary Optic Neuropathy (LHON) and Dominant Optic Atrophy are the most frequent inherited optic neuropathies. LHON is the most common maternally inherited disease linked to mitochondrial DNA (mtDNA). The patients present with subacute asymmetric bilateral vision loss. LHON is characterized by incomplete penetrance - most maternally related individuals who carry a mtDNA mutation do not develop optic neuropathy [**2**]. The three most common mtDNA mutations responsible for 90-95% of LHON cases are m.3460G>A (*MTND1*), m.11778G>A (*MTND4*), and m.14484T>C (*MTND6*) mutations, but further rare mtDNA mutations are also described as pathogenic for LHON [**3**]. Only 50% of men and 10% of women who carry the LHON mtDNA mutation develop optic neuropathy [**4**]. The presence of pathogenic mutations does not always correlate with vision loss.

Males aged between 20 and 30 years old are typically affected. Patients present with acute or subacute painless central vision loss in one eye. Within weeks, the second eye is also affected, the average interval time being 6 weeks. A few months after onset, the vision acuity will stabilize at or below 20/200 [**5**]. 

In the acute phase of LHON, the fundus exam shows hyperemia of the optic disc and peripapillary microangiopathy, but no leakage. Central or cecocentral scotomas are another typical finding. The chronic phase is reached 12 months after onset. 

## Case report

We present a case of a 28-year-old man who came to our clinic complaining of a subacute decrease in visual acuity (VA) in his left eye. From his medical history, we found out that 1 month before he had the same symptoms in the right eye, but neglected them. From the family history, we noted that an uncle has had vision problems since childhood. Personal history was insignificant. The clinical exam was within normal limits.

**Fig. 1:**
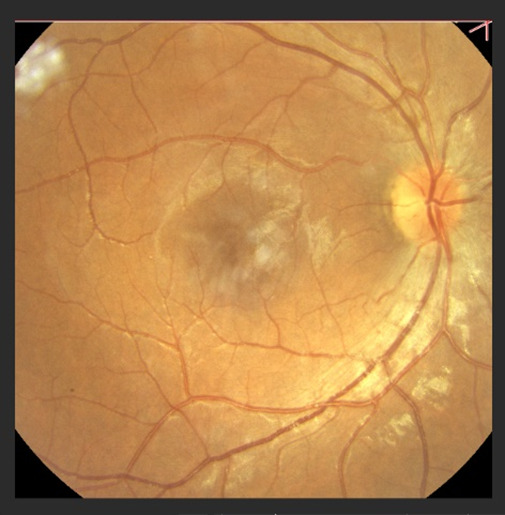
Fundus right eye

**Fig. 2:**
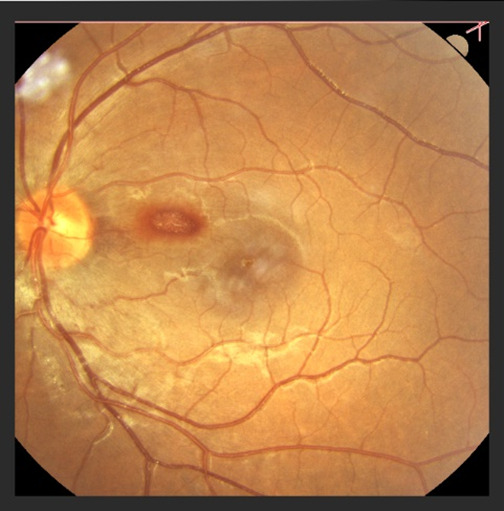
Fundus left eye

On the ophthalmic exam, we found the best corrected VA right eye (RE) 1/50 and 7/10 for the left eye (LE). Slit lamp examination of the anterior segment of the eye was within normal limits. The fundus for both eyes was examined and revealed congestive, prominent optic discs, with narrow vessels and macular reflex (**Fig. 1**,**2**). 

**Fig. 3:**
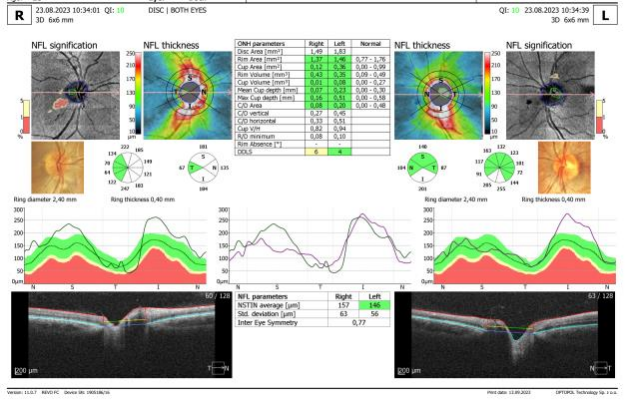
OCT - Optic nerve oedema RE and LE

**Fig. 4:**
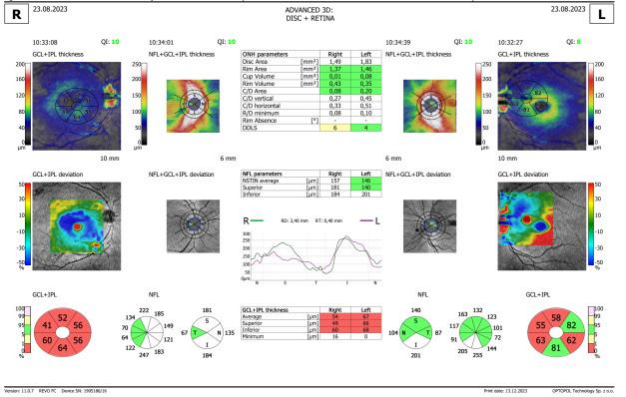
OCT - GCL thinning RE and LE

Spectral-domain Optical coherence tomography (SD-OCT) revealed a swollen optic nerve, thickening of the peripapillary retinal nerve fiber layer (pRNFL) in the superior, nasal, and inferior quadrants for the RE and inferior quadrant LE, and a thinning in the retinal ganglion cells layer (GCL) (**Fig. 3**,**4**). 

The color vision test showed a dyschromatopsia on red-green axes. 

**Fig. 5:**
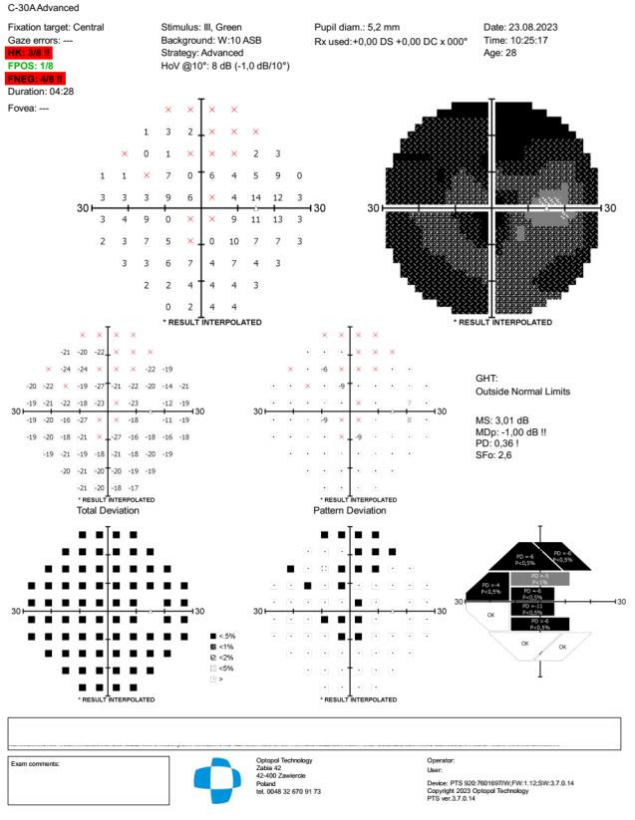
Visual field RE

**Fig. 6:**
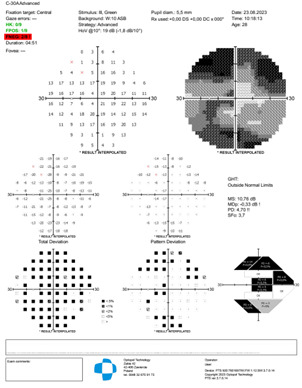
Visual field LE

The visual field Optopol showed a decrease in general retinal sensitivity and a central scotoma (**Fig. 5**,**6**).

First, our patient had a magnetic resonance imaging (MRI) exam, as we wanted to exclude a neurosurgical problem. The MRI showed bilateral optic nerve swelling: in the axial sequence 3 D, the optic nerves had similar thickness, retrobulbar 6.5 mm - normal 1.5-4.4 mm; symmetrical enlargement of intergyral grooves, mainly parieto-occipital; no pathological contrast fixation and no demyelinating cerebral lesions in the cervical spine were observed.

The visual evoked potentials (VEP) showed an increase in 60ʹ and 15ʹ VEP implicit time P100 (VEP IT) - 125,9 msec. and a reduction in 60ʹ and 15ʹ VEP amplitude N75-P100 (VEP A) - 3,62 µV.

The initial differential diagnosis included infectious causes, autoimmune diseases, demyelinating pathologies, and hereditary optic neuropathies. We carried out complete blood tests, including complete blood count, and specific antibodies for autoimmune and infectious diseases. Laboratory tests were within normal limits. We excluded an infectious cause, as the Ab against Borrelia burgdorferi, Toxoplasma, Treponema pallidum (TPHA/RPR), and Bartonella henselae were negative. The tuberculin intradermal reaction and the thorax X-ray were within normal limits. We started treatment with a high dose of corticosteroid, intravenous methylprednisolone sodium succinate 1g daily for 3 days, followed by oral prednisolone 1 mg/kg/day for 11 days, and then tapered for 3 days. We did not observe any improvement in visual acuity. 

The Ab anti-Aquaporine 4, specific for Neuromielitis optica DeVic came negative, and the neurological consult was normal. A blood test of the mtDNA showed the presence of 11778 G>A mutation on the mtND6 gene.

The medical history, the fundus appearance, the OCT, and the paraclinical investigations, made us diagnose our patient with Leber’s hereditary optic neuropathy (**Table 1**). 

**Table 1:** The differential diagnosis of LHON vs. optic neuritis

Red flags suggestive of LHON	Red flags suggestive of optic neuritis
Male (80% of cases)	Female (65% of cases)
Painless	Periocular pain with eye movement
Subacute loss of vision - worsening symptoms for 4-6 weeks per eye	Acute loss of vision - worsening symptoms up to 2 weeks
No spontaneous recovery	Spontaneous recovery within 2-3 weeks
Bilateral vision loss - sequential	Unilateral vision loss
Pupillary reflexes preserved	Relative afferent pupillary defect
Absence of retinal disc leakage on fluorescein angiography	Retinal disc leakage may be present on fluorescein angiography
No/few signs of multiple sclerosis	There may be other signs suggestive of multiple sclerosis
MRI findings not typical of multiple sclerosis (no CNS lesions)	MRI findings typical of multiple sclerosis (CNS lesions)
Family history of LHON symptoms, or confirmed carriers within the family	No family history of LHON

We initiated treatment with systemic idebenone, 900 mg/day - 6 tablets/day. We examined the patient after 2, 6, and 10 weeks of treatment. The recommendation was that the patient had to be followed monthly in the first 3 months and after that, every 3 months. Monitoring consisted of an examination of VA, fundus, and OCT, color perception, and visual field.

**Fig. 7:**
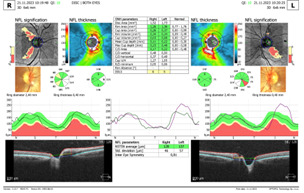
OCT of optic nerve head

**Fig. 8:**
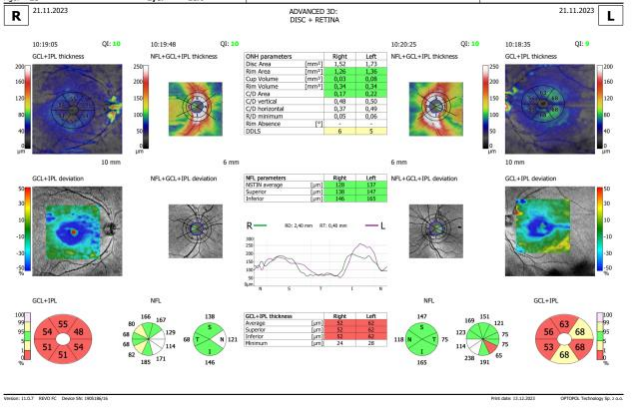
Thinning of the retinal ganglion cell layer

The ophthalmological exam after 2 weeks of treatment showed a VA of 2/50 RE and 5/10 LE, with the same fundus appearance and OCT measurements. After 6 weeks of treatment, we observed a decrease in VA in both eyes, VARE 1/100 and VALE 1/10, accompanied by a slightly pale optic nerve head, in the temporal quadrant and an increase in atrophy in the retinal ganglion cell layer LE on OCT exam (**Fig. 7**,**8**). The pRNFL showed a thinning mostly in the temporal and inferior quadrants.

After 10 weeks of treatment, we noticed a stabilization of VA, fundus, and OCT measurements, and the patient reached the nadir. The VARE was 1/100 and VALE 1/10. He tolerated well the treatment with idebenone and had no side effects.

## Discussions

Leber’s hereditary optic neuropathy (LHON), first reported by Theodore Leber in 1871, has been considered an example of maternal inherited disease linked to mitochondrial mutations. The onset is most frequently in the second or third decade of life, but manifestations in childhood or at an older age have been reported. About 40% of the patients with LHON do not have a clear family history [**5**]. The mtDNA mutation alone is not sufficient to determine the disease in a carrier. Environmental risk factors, which include tobacco, alcohol, and agricultural pesticides are believed to play an important role [**6**]. In LHON-affected patients, mutations result in the dysfunction of the mitochondrial respiratory chain complex I, which leads to increased reactive oxygen species [**1**]. Approximately 95% of the LHON cases are caused by m.3460G>A (*MTND1*), m.11778G>A (*MTND4*), and .14484T>C (*MTND6*) mutations [**3**]. Stenton et al. reported that certain variants in the nuclear gene *DNAJC30* result in an autosomal recessive inherited form of LHON and a strong geographic accumulation of this gene was reported in Eastern Europe [**7**].

The hallmark of hereditary optic neuropathies determined by mitochondrial dysfunction is the vulnerability and degeneration of retinal ganglion cells [**5**]. In the acute phase, the optic discs appear hyperemic, with a characteristic circumpapillary telangiectatic microangiopathy. The RNFL is swollen without evidence of leakage of dye on fluorescein angiography. At the early stages, RGC and their axons in the papillomacular bundle are the most susceptible [**8**]. Over time, axonal loss leads to disc pallor and diffuse optic atrophy. Best visual acuity in the chronic stage nearly always decreases to 20/200 or below and large central scotomas are seen on the visual field [**9**]. 

OCT is an important tool in monitoring patients diagnosed with LHON. OCT measures the RNFL and RGC changes at different phases, which helps find out a specific pattern of the natural history of LHON. In recent years, studies found that the VEP amplitude of P100 and the pRNFL thickness were reduced in individuals affected by LHON, which indicates the impaired function and structure of RGC in these patients [**10**,**11**].

In our patient, a significant increase in pRNFL thickness was detected in the inferior quadrant, in the acute phase. This is consistent with other studies. Barboni et al. showed that the pRNFL thickness increase first appeared at the temporal and inferior quadrants. This is in correlation with the early involvement of the papillomacular bundle. They also found that the late involvement of superior and nasal quadrants suggests a dynamic evolution that continues for 3 months. This may represent a therapeutic window of opportunity [**12**]. There are still controversies regarding the way pRNFL thickness changes in the subacute phase. Wang et al. found that the average pRNFL thickness of the affected patient with disease duration less than 3 months did not change significantly over follow-up [**13**,**11**]. Tian et al. found that the superior pRNFL thickness in patients with a disease duration of less than 3 months was increased compared to the controls [**14**]. The reasons behind pRNFL thickening are still controversial. RNFL thickening might be correlated with axonal edema and mitochondrial redistribution at dysfunctional ganglion cells [**15**]. 

Regarding the RGC, we observed a decrease in thickness, first seen in our patient's temporal quadrant (LE). This decrease could be due to the involvement of the papillomacular bundle at the time of onset because it had a small diameter. Later, all the quadrants were affected (RE). Several studies found that in addition to pRNFL thickness, the macular RGC thickness of the affected eye was also significantly decreased [**11**,**13**,**16**]. Balducci et al. have reported that the macular ganglion cell layer and inner plexiform layer (GC-IPL) become significantly thinner before the appearance of any symptom [**17**].

VEP responses measured in our patient were obtained by using different spatial frequencies, subtending respectively 60 minutes and 15 minutes of visual angle, to obtain information on the function of both large and small axons forming the visual pathways. VEP implicit time showed a delay P100 at 60ʹ and 15ʹ and a reduction in amplitude N75-P100 at 60ʹ and 15ʹ. Parisi et al. showed significant abnormal VEP responses in all LHON eyes when compared with controls. In their study, RGC and visual pathways function were, on average, not statistically modified through 12 months of follow-up of the chronic phase of the disease [**18**]. Our results agree with those reported by Ziccardi et al. and Yang et al., who considered VEP parameters in the recruitment for gene therapy and their changes in evaluating its effects [**19**,**20**].

In our patient, the OCT exam showed a normal-sized optic disc. The anatomic conformation of the optic nerve head presented variability in the general population, as measured by different techniques. The optic disc conformation might be a factor that influences both penetrance and clinical expressivity. Sadun et al. hypothesized that the “disc at risk”, which was studied in ischemic optic neuropathies, may also play a role in the pathogenesis of LHON [**21**]. Ramos suggested that a larger disc may be a protective factor, as it is associated with less crowding of RGC axons. Thus, larger discs prevent carriers from developing the acute phase. In their study, the affected subgroup of patients with visual recovery had a larger optic nerve head [**22**]. Another study that included a group of children with LHON, with the onset of the disease before 10 years, showed a significantly smaller optic disc area in these children. This suggests that small optic discs may represent an unfavorable prognostic factor [**23**].

The MRI in our patient showed that the mean values of optic nerve volumes were higher than in healthy controls, which might be correlated with axonal edema and mitochondrial redistribution in the acute phase.

Unfortunately, a definitive treatment for LHON does not exist, but some options are currently available and under continuous evolution. LHON mutations variably affect Complex I, resulting in reduced efficiency in the synthesis of ATP. While searching for new efficient therapies, one should consider the variability of symptoms and the possibility of spontaneous visual recovery among these patients. We should not forget that in a variable number of LHON patients (especially with the 11778/ND4 mutation), there is the possibility that worsening or improvement of visual function can occur spontaneously during the disease’s natural history. Timing is an important aspect. Scientists agreed that subacute and acute phases of the disease should be the target for early treatment, to prevent further vision loss. The “sooner the better” paradigm is the key to clinical practice [**17**,**24**].

The only molecule approved since 2015 for LHON is idebenone, a synthetic analogue of CoQ10 that shares its antioxidant properties. Idebenone is thought to partly mitigate oxidative stress. The conversion into the reduced form allows idebenone to shuttle the electrons directly to complex III, bypassing the complex I dysfunction occurring in LHON, thus, preserving energy production [**25**]. The efficacy of idebenone in LHON patients was assessed in a randomized, double-blinded, placebo-controlled study, conducted by Klopstock in 2011. The endpoints were changes in best-corrected VA and changes in VA of the best eye at baseline and for both eyes independently. Results showed that idebenone was safe and well tolerated. The results showed a consistent positive trend in treated patients compared to controls, whose VA deteriorated [**26**]. In their study, Carrelli et al. showed that the proportion of patients who recovered from VA was higher among the treated group and that visual improvement was correlated with early treatment and a longer duration of therapy [**27**]. 

In 2015, idebenone (Raxone®) was granted market authorization for the treatment of LHON in the European Union. Experts agreed that idebenone treatment at 900 mg/day should be started as soon as possible in the acute stage [**24**]. Lifestyle counseling is also very important, especially about quitting smoking and alcohol consumption.

We did not observe an improvement in VA, in our patient, even if we started the treatment early in the acute phase. The VA probably decreased until it reached the nadir and we hoped for a better improvement in the following months. Long-term follow-up data of LHON patients treated with idebenone have been collected through an Expanded Access Programme. They observed a visual improvement in 46.0% of LHON-treated patients. The time to initial visual recovery varied between 10 weeks and 26.5 months, with a mean period of 9.5 months. These results sustain the importance of finding a consensus for the length of treatment administration, to maximize its effect [**28**].

The goal for the treatment of LHON is the correction of the genetic defect and the prevention of its transmission. The eye is easily accessible by intravitreal injection of appropriate viral vectors. That is why it represents a suitable target for gene therapy, to correct gene defects [**29**]. Guy et al. assessed the safety and tolerability of an adenovirus-associated vector expressing a normal ND4 complementary DNA. In their study, at 12 months follow-up, 66% of treated eyes had an improvement in VA of 3 lines or more. No injected eyes lost 3 lines of VA, while 22% of eyes in the natural history group at baseline lost more than 3 lines [**30**]. The allotopic gene therapy targets the RGC by intravitreal injections. The combination of idebenone and gene therapy may result in a synergistic and complementary therapeutic effect [**31**]. 

## Conclusions

The differential diagnosis of optic nerve edema may be challenging, as it may have many causes, from demyelination to infectious and autoimmune, but we should also keep in mind the hereditary optic neuropathies. Due to genetic tests that allow genome sequencing, the diagnosis of LHON should be considered more frequently.

The treatment is under continuous evolution; it ranges from drugs designed to compensate the mitochondrial dysfunction, to stem-cell and gene therapies. Timing is a crucial aspect, the “sooner the better” paradigm is the key in clinical practice. It is important to remember that it is possible that the worsening or the improvement of visual function in a variable number of LHON patients can spontaneously occur during the disease’s natural history.

**Conflict of Interest**

The authors have no conflict of interest.

**Informed Consent and Human and Animal Rights Statement**

A written, informed consent was obtained from the subject, to share the information, and medical records, including pictures of the patient. 

**Authorization for the use of human subjects**

Ethical approval: The research related to human use complies with all the relevant regulations and institutional policies, is by the tenets of the Helsinki Declaration, and has been approved by the review board of “N. Oblu” Clinical Emergency Hospital, Iași, Romania.

**Acknowledgments**

None.

**Sources of Funding**

None.

**Disclosures**

None.
